# Deep sequencing is an appropriate tool for the selection of unique Hepatitis C virus (HCV) variants after single genomic amplification

**DOI:** 10.1371/journal.pone.0174852

**Published:** 2017-03-31

**Authors:** Thibault Guinoiseau, Alain Moreau, Guillaume Hohnadel, Nicole Ngo-Giang-Huong, Celine Brulard, Patrick Vourc’h, Alain Goudeau, Catherine Gaudy-Graffin

**Affiliations:** 1 INSERM U966, Université François Rabelais and CHRU de Tours, Tours, France; 2 Institut de Recherche pour le Développement (IRD) UMI 174 PHPT-Faculty of Associated Medical Sciences, Chiang Mai University, Chiang Mai, Thailand; 3 UMR INSERM U930, Université François Rabelais, Tours, France; University of Cincinnati College of Medicine, UNITED STATES

## Abstract

Hepatitis C virus (HCV) evolves rapidly in a single host and circulates as a quasispecies wich is a complex mixture of genetically distinct virus’s but closely related namely variants. To identify intra-individual diversity and investigate their functional properties *in vitro*, it is necessary to define their quasispecies composition and isolate the HCV variants. This is possible using single genome amplification (SGA). This technique, based on serially diluted cDNA to amplify a single cDNA molecule (clonal amplicon), has already been used to determine individual HCV diversity. In these studies, positive PCR reactions from SGA were directly sequenced using Sanger technology. The detection of non-clonal amplicons is necessary for excluding them to facilitate further functional analysis. Here, we compared Next Generation Sequencing (NGS) with *De Novo* assembly and Sanger sequencing for their ability to distinguish clonal and non-clonal amplicons after SGA on one plasma specimen. All amplicons (n = 42) classified as clonal by NGS were also classified as clonal by Sanger sequencing. No double peaks were seen on electropherograms for non-clonal amplicons with position-specific nucleotide variation below 15% by NGS. Altogether, NGS circumvented many of the difficulties encountered when using Sanger sequencing after SGA and is an appropriate tool to reliability select clonal amplicons for further functional studies.

## Introduction

Hepatitis C virus (HCV) is an enveloped positive sense single stranded RNA virus of 9600 bases, which infects 130–150 million people worldwide. Most (70%) HCV infections become chronic and progress toward liver diseases such as cirrhosis and hepatocellular carcinoma[1]. Approximately 500 000 people die each year from hepatitis C-related liver diseases[2].

*In vivo*, HCV replicates rapidly using a viral RNA polymerase that lacks proofreading activity[3]. The error rate of HCV polymerase has been estimated *in vitro* to be 10^−3^ nucleotide substitutions per site per year[4]. This high mutation rate combined with a short generation time (10^12^ virions produced per day[5]) is at the origin of the quasispecies dynamics of RNA viruses[6]. Seven genotypes have been described, which differ by 30 to 35% in their nucleotide sequence[7]. HCV circulates in infected individuals as a complex mixture of genetically different, but closely related, viral variants[8], [9]. Constraints in the viral genome and protein structure prevent some variants from proliferating[10]. Rapid HCV evolution in a single host favors the emergence of mutants that can escape from specific immunity[11].

Investigating the functional properties of intra-individual HCV variants *in vitro* requires accurate identification of HCV variants within a quasispecies. Single genome amplification (SGA) can be used in this context. It consists of serially diluting cDNA to amplify single cDNA molecules (clonal amplicon). However, the amplification of two or more cDNA molecules (non-clonal amplicon) cannot be fully ruled out. Although initially developed for the study of HIV quasispecies diversity[12]^,^[13], SGA has also been used to describe early diversification of HCV after transmission events[14]^,^[15]^,^[16], [17]^,^[18]. In those studies, conventional Sanger sequencing technique was performed on positive PCR reactions coming from SGAs. Visual inspection of electropherograms is required for detecting mixed populations. Non-clonal amplicons must be excluded to achieve the most accurate representation of the variant population. This is paramount for performing further functional analyses to study, for example, the transmission of variants from mother to child using *in vitro* models, such as HCV retroviral pseudoparticles (HCVpps)[19] or infectious hepatitis C virus coming from cell culture (HCVcc) [20]. Another limitation of conventional Sanger sequencing is the necessity to use specific sequencing primers. This represents a major challenge, especially in variable regions such as the HCV E1E2 gene, and could compromise the sequencing of long fragments.

Here, we compared Next Generation Sequencing (NGS) with *de novo* assembly to the classical Sanger approach with the aim of improving the differentiation between clonal and non-clonal amplicons after SGA.

## Materials and methods

### Biological material

A plasma specimen was obtained in the year 2000 from a Thai woman infected by the HCV 1b genotype. She was participating in HIV prevention clinical trial that assessed different duration of maternal and infants zidovudine for the prevention of perinatal transmission of HIV[21]^,^[22]. The HCV genotype was determined by NS5B amplification and sequencing.

### Viral RNA extraction and cDNA synthesis

Viral RNA was extracted from 400 μL plasma using the Macherey Nagel NucleoSpin Virus kit (Macherey Nagel, Hoerdt, France). The RNA was eluted and immediately reverse-transcribed using random hexamer primers and Superscript III kit (Invitrogen, Life technologies, Courtaboeuf, France) to generate cDNA according to the manufacturer’s protocol.

### Single genome amplification of full-length E1E2 glycoproteins

Full-length E1E2 glycoprotein genes were amplified using an SGA approach. A series of cDNA dilutions (1:40, 1:80, 1:100, 1:150, 1:200) was amplified by nested PCR, resulting in a fragment of 2097 base pairs. We selected the dilution giving a maximum of 30% positive PCR reactions (dilution 1:40)[12,17]. Indeed, according to the Poisson distribution law, a majority of wells at this dilution contains a single cDNA molecule. PCR amplification was carried out using high fidelity Platinum Taq PCR SuperMix (Invitrogen Life technologies, Courtaboeuf, France) according to the manufacturer’s protocol. The PCR primers for generating the full-length E1E2 glycoproteins sequences were: first-round sense primer P1bE1E2extsens (5’-ACCAAACGTAACACCAACCGC-3’; position 372 to 392, H77), first-round antisense primer P1bE1E2extantisens (5’-GCTCTGGTGATAAAATATTGTAACCAC-3’; position 2873 to 2899, H77), second-round sense primer P1bE1E2intsens (5’-TGGGTAAGGTCATCGATACCCT-3’; position 697 to 718, H77), and second-round antisense primer P1bE1E2intantisens (5’-CACGATGCAGCCATCTCCCG-3’; position 2775 to 2794, H77). The PCR amplification conditions were: 94°C for 2 min followed by 35 cycles of 94°C for 30 s; 58°C for 30 s; 68°C for 2 min 30 s; before a final extension at 68°C for 10 min. The product of the first PCR was used as a template for the 2^nd^ PCR under the same conditions. The PCR products were separated by microfluidics capillary electrophoresis (LabChip GX–Perkin Elmer). Only PCR products obtained at the dilution giving a maximum of 30% positive PCR reactions are selected.

### DNA sequencing using NGS

**Fig 1:**
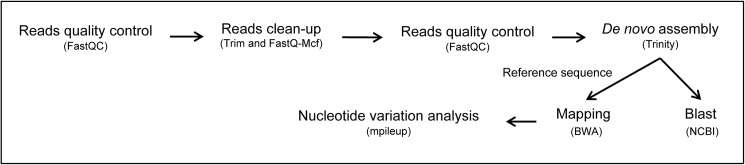
Workflow of analysis on Galaxy platform.

The sequencing library was built using a Nextera XT DNA sample preparation kit (Illumina, San Diego, USA) according to the manufacturer’s protocol. 1 ng of DNA per sample is necessary. This protocol includes: tagmentation of both DNA strands, a PCR amplification step that adds adapters and indexes, and a clean-up step using AMPure XP beads (Agencourt—Beckman Coulter, Roissy, France). Finally, a beads-based normalization of each library according to the manufacturer’s instructions was performed to ensure equal library representation in the pooled sample. Heat-denaturation of the library pool was performed before the sequencing run. Paired-end sequencing of 151 base-pairs was performed on a Miseq (Illumina, San Diego, USA) platform. Illumina sequencer output files matching 151 base-pair sequencing reads were processed using the “Biomina Galaxy platform”[23] after verifying read quality (FastQC algorithm). *De novo* assembly was performed using the Trinity program. The constructed sequences were verified using the Blast option of the National Center for Biotechnology Information (NCBI). Single reads with a QC score over 30 and a length of over 30 nucleotides were conserved for *de novo* assembly using the Trinity program. Reads were then mapped using the “Burrows-Wheeler Aligner” (BWA) to the reference sequence given by the de novo assembly. Nucleotide analysis was performed position by position using the mpileup program (Fig 1). Positions with a sequencing depth of over 100X were retained for further analysis. Non-clonal samples (ie amplicons containing multiple templates) were identified by determining nucleotide heterogeneity, position by position.

15 to 30% of position had some variability due to the NGS technique. We subjected a known clonal sample from an RNA virus to the deep sequencing to distinguish variability due to background from that due to non-clonal differences. Thus, we could determine that a clonal sample contained a nucleotide variation of less than 5% or 50 nucleotides at each position. Non-clonal samples were defined as those having a nucleotide variation of more than 10% or 100 nucleotides at one or several positions. Ambiguous samples had a nucleotide variation of between 5 and 10% or 50 and 100 nucleotides at one or several positions.

### DNA sequencing using Sanger method

**Fig 2:**
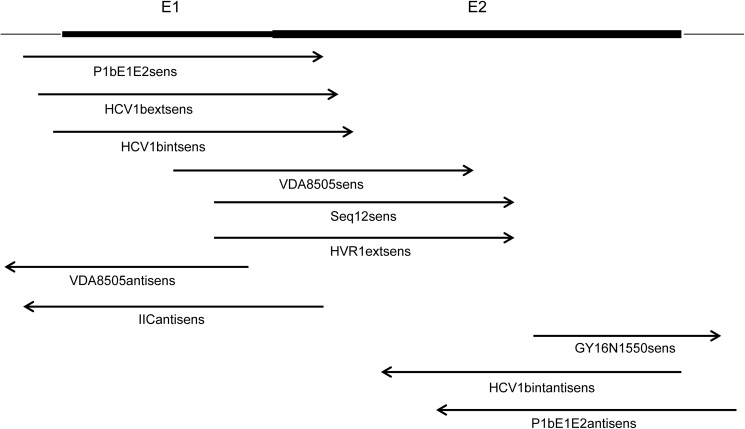
Overlapping primers for Sanger sequencing.

The selected samples from SGA were also sequenced using BigDye terminator chemistry, on an ABI 3130xl capillary sequencer (Applied Biosystems—Life Science Technologies, Carlsbad, USA). According to the manufacturer’s instructions, 10 ng of DNA was used for each sequencing reaction. The sequencing primers were designed using vector NTI® (Thermo Fisher Scientific, Courtaboeuf, France) based on the consensus sequences obtained following the Miseq de novo assembly (Fig 2).

During a preliminary analysis (performed with Sequencing Analysis software™), the threshold for second peak height was set at 25% of the main peak. A secondary step of analysis was needed for the final validation of second peaks (performed with CLC Main Workbench^TM^). It was based on visual inspection of electropherograms especially in homopolymer regions. Background signal as well as alignments of electropherograms obtained with different primers, enable to validate or reject second peak.

Non-clonal samples were identified by identifying double peaks on electropherograms position by position.

### Primer for Sanger sequencing

The primers used for Sanger sequencing are shown in Table 1.

**Table 1:** Primers for Sanger sequencing.

Primer	Location*	Sequence (5'-3')	Samples†
P1bE1E2sens	697–718	TGGGTAAGGTCATCGATACCCT	All except c14, c16, c17
HCV1bextsens	821–841	CGGCGTGAACTATGCAACAGG	All except c3, c14, c15, c16, c17, c37
HCV1bintsens	843–873	CACCATGGGTTGCTCTTTCTCTATCTTCC	c6, c12, c20, c27, c37, c42
VDA8505sens	1178–1196	CCTGTGCGGGTCGTTTTT	All except c14, c16, c17
seq12sens	1290–1309	CGCATGGCTTGGGATATGAT	c34, c36
HVR1extsens	1290–1310	CGCATGGCGTGGGACATGATG	c15
VDA8505antisens	1435–1454	AACCTTAGCCCAGTTCCCCG	All except c14, c16, c17
IICantisens	1605–1620	ACCCAGTGCTATTCAT	All except c14, c15, c16, c17, c37, c42
GY16N1550sens	2247–2266	GTGGAGCACAGGTTCGAAGC	All except c14, c16, c17, c37, c41
HCV1bintantisens	2559–2584	AATCAGGCCTCAGCCTAGGCTATCTG	c12, c20, c27, c37, c42
P1bE1E2antisens	2775–2794	CACGATGCAGCCATCTCCCG	All except c14, c16, c17

* Location indicate nucleotide postion relative to the H77 genome

† Samples sequencing with each primer

### Nucleotide sequence accession numbers

The clonal sequences contigs and the reads from the NGS sequencing were deposited in GenBank under the accession numbers KX358443 to KX358470.

### Ethics statement

The parent studies received initial and annual approvals by the Thai Ministry of Public Health Ethics Committees and other relevant Ethics committees.

All participants or their guardians provided their informed consent to participate in the parent studies. Their consent includes explicitly the use of the blood/plasma samples in other future studies.

## Results

### NGS and Sanger sequencing

Forty-two positive PCR products issued from SGA fulfilled the criteria of PCR selection process (ie PCR products obtained at the dilution giving a maximum of 30% positive PCR reactions). were purified for sequencing.

We first sequenced the HCV E1E2 amplicons derived from SGA by NGS. In total, 42 PCR products (samples), identified c1 to c42, were successfully sequenced and analyzed. The sequencing depth varied between samples, but was between 100x (our threshold) at the end of fragments and 6545x in the middle of the fragments. We classified 28 samples as clonal, 11 as non-clonal and three as ambiguous.

The same 42 samples were also sequenced by Sanger technology using 6 to 11 primers based on the consensus sequences as described before. Samples c14, c16, and c17 could not be sequenced because of an insufficient quantity of DNA. After analysis of the electropherograms of the remaining 39 samples, we classified 32 samples as clonal and 7 as non-clonal.

### Assignment of clonal and non-clonal samples: Comparison between NGS and Sanger sequencing

We compared the assembled sequences obtained with NGS and those obtained with Sanger sequencing (Table 2). All 28 samples classified as clonal by NGS were also classified as clonal after direct Sanger sequencing. Of the three samples classified as ambiguous by NGS, two were considered to be clonal after direct Sanger sequencing, and the remaining one non-clonal. Among the 11 samples classified as non-clonal by NGS, six were also classified as non-clonal after direct Sanger sequencing and two was re-assigned to the clonal category; three samples could not be sequenced. These discrepancies in the classification of samples were probably due to the depth of the NGS sequencing at each position for the nucleotide variation analysis. The sequencing depth for all positions was at least 100X and nucleotide variations at each position are clearly indicated by the software. This is less subjective than interpreting electropherograms following direct Sanger sequencing.

**Table 2:** Comparaison of clonality sample using two different sequencing technology.

Sample	MiSeq sequencing	Sanger sequencing	Agreement
c1	C	C	●
c2	A (0*; 2†)	C	○
c3	NC (1; 1)	NC (1‡; 0§)	●
c4	NC (3; 0)	A (0; 3)	○
c5	NC (3; 2)	NC (4; 0)	●
c6	C	C	●
c7	A (0; 1)	C	●
c8	C	C	●
c9	C	C	●
c10	C	C	●
c11	C	C	●
c12	A (0; 3)	NC (2; 0)	○
c13	C	C	●
c14	NC (2; 0)	///	◊
c15	NC (2; 2)	A (0; 1)	○
c16	NC (1; 0)	///	◊
c17	NC (7; 0)	///	◊
c18	C	C	●
c19	C	C	●
c20	C	C	●
c21	C	C	●
c22	C	C	●
c23	C	C	●
c24	C	C	●
c25	C	C	●
c26	NC (1; 0)	NC (1; 0)	●
c27	C	C	●
c28	C	C	●
c29	C	C	●
c30	C	C	●
c31	NC (1; 0)	C	○
c32	C	C	●
c33	C	C	●
c34	C	C	●
c35	C	C	●
c36	C	C	●
c37	C	C	●
c38	NC (1; 0)	C	○
c39	C	C	●
c40	C	C	●
c41	NC (2; 0)	NC (2; 0)	●
c42	C	C	●

* number of non clonal position with MiSeq sequencing analysis.

† number of ambiguous postion with MiSeq sequencing analysis.

‡ number of non clonal position with Sanger sequencing analysis.

§ number of ambiguous postion with Sanger sequencing analysis.

● MiSeq and Sanger are in agreement.

○ MiSeq and Sanger are in desagreement.

◊Failure of Sanger sequencing.

To check our results, 6 amplicons (positive after SGA) were selected to be sequenced a second time with the two techniques. This relates to all samples in disagreement between NGS and Sanger sequencing (c2, c7, c31 and c38) and two in agreement (c12 and c41).

The sequencing results (NGS and Sanger sequencing) are similar to our previous results (data not shown).

**Fig 3:**
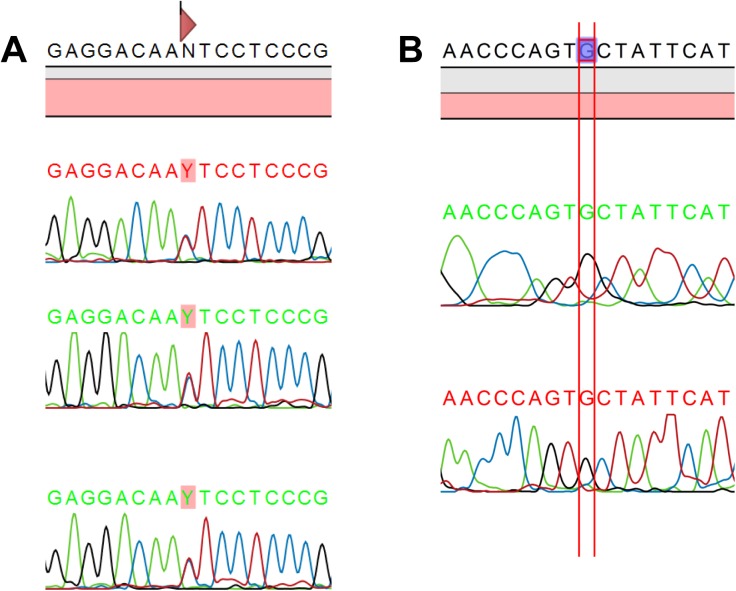
Examples of superimposed electropherograms. (A) in agreement with NGS (sample c41; 48,3% nucleotide variation; double peak seen on electropherograms) (B) in disagreement with NGS (sample c38, 10,5% nucleotide variation; absence of double peak on electropherograms).

We compared the percentage of nucleotide variation observed after NGS analysis at each non-clonal and ambiguous position with the electropherogram profiles obtained by Sanger sequencing. Double peaks were clearly visible on electropherograms for position-specific nucleotide variation of above 35% by NGS. Double peaks were less visible and mixed bases could be easily missed for 15–20% nucleotide variation by NGS, and no double peaks were visible on the electropherograms for a nucleotide variation of less than 15% by NGS (Table 3). Two examples of electropherograms are presented in Fig 3.

**Table 3:** Comparaison of non clonal position with two sequencing techniques according to the percentage of MiSeq nucleotide variation.

MiSeq sequencing								Sanger sequencing	
Sample	Location*	Depth	A	C	G	T	% variation^†^	Double peak	Primers^‡^
c41	1028	1759	1	819	0	878	48,3	"+"	3
c5	1380	3085	1821	0	1264	0	41	"+"	3
c5	916	1937	1147	0	790	0	40,8	"+"	3
c5	2048	3202	0	1992	0	1210	37,8	"+"	2
c3	1431	2523	910	0	1612	1	36,1	"+"	2
c26	1973	1803	626	0	1177	0	34,7	"+"	2
c4	1707	2745	2217	0	528	0	19,2	"+/-"	2
c4	1670	2302	0	421	0	1881	18,3	"+/-"	2
c15	905	1479	1237	0	0	242	16,4	"+/-"	2
c4	2168	2630	400	0	2230	0	15,2	"+/-"	2
c31	2379	2387	2069	0	318	0	13,3	"-"	2
c38	2012	1571	0	1406	0	165	10,5	"-"	2
c15	1615	1343	0	1236	0	107	8	"-"	1
c41	2479	2952	0	2726	0	226	7,7	"-"	1

* Location indicate nucleotide postion relative to the H77 genome.

† % nucleotide variation at this postion after MiSeq analysis.

‡ Number of nucleotide read at this position according to the numbers of primers used.

"+" Double peak clearly visible on electrophoregram at this position.

"+/-" Double peak no clearly visible on electrophoregram at this position.

"-" No double peak on electrophoregram at this position.

For nucleotide variations below 7.4% (i.e. at ambiguous positions), the electropherogram profiles were very heterogeneous with double peaks visible in some but not in others (Table 4).

**Table 4:** Comparaison of ambiguous position with two sequencing techniques according to the percentage of MiSeq nucleotide variation.

MiSeq sequencing								Sanger sequencing	
Sample	Location*	Depth	A	C	G	T	% variation^†^	Double peak	Primers^‡^
c15	1008	1114	1032	0	82	0	7,4	"-"	1
c12	2082	1736	0	0	80	1656	4,6	"+"	2
c2	826	1615	0	68	0	1547	4,2	"-"	3
c15	1322	1681	1617	0	64	0	3,8	"-"	1
c12	1858	1723	0	62	0	1661	3,6	"-"	1
c12	2291	2104	70	0	2034	0	3,3	"+"	1
c5	1274	1856	0	1798	0	58	3,1	"+"	4
c3	2191	3735	3636	0	99	0	2,7	"-"	2
c5	1811	3209	3129	0	80	0	2,5	"-"	2
c10	1603	2531	2472	0	59	0	2,3	"-"	2
c2	2558	3174	72	0	3102	0	2,3	"-"	3

* Location indicate nucleotide postion relative to the H77 genome.

† % nucleotide variation at this postion after MiSeq analysis.

‡ Number of nucleotide read at this position according to the numbers of primers used.

"+" Double peak clearly visible on electrophoregram at this position.

"+/-" Double peak no clearly visible on electrophoregram at this position.

"-" No double peak on electrophoregram at this position.

## Discussion

Exploration of the *in vitro* functional properties of HCV variants circulating in a single host requires accurate determination of the HCV quasispecies sequences. SGA followed by Sanger sequencing has been used for this purpose by several groups. However, direct sequencing has several limitations that we aimed to circumvent using NGS. Bioinformatics analysis after NGS sequencing is clearly more complex than Sanger sequencing but our work was facilitated by the possibility to design a convenient workflow using the Galaxy Biomina platform.

We were able to entirely sequence envelope genes of all 42 HCV samples (approximately 2000 nt) without any failures using the Illumina NGS technology (Table 2). Only 1 ng of DNA per sample was necessary for NGS whereas a total amount of 60 ng was necessary for Sanger sequencing: 10 n g for each of the six primers (480 Sanger sequencing PCR reactions were necessary for the 42 samples). Finally, three sequences could not be obtained (Table 1), because of the limited quantity of the amplified DNA.

NGS Sequencing has obvious advantages because it spares DNA and enables further plasmid construction for functional *in vitro* analyses. The use of non-specific sequencing primers for Illumina NGS sequencing provides a clear benefit. DNA is simultaneously fragmented and tagged with sequencing adapters. The HCV E1/E2 gene exhibits a high rate of genetic polymorphism, making it challenging to choose a reference sequence and design specific primers. In NGS analysis, no reference sequence is required. The reads are assembled as contigs and the coverage quality of the *de novo* consensus sequence depends only on the size and continuity of the contigs. This *de novo* consensus sequence can then be used to choose adequate primers for Sanger sequencing. This strategy has clearly reduced the sequencing failures encountered using Sanger sequencing, but may have introduced a potential bias in our comparison.

All samples classified as clonal after NGS were also classified as clonal after Sanger sequencing. According to the Poisson distribution, cDNA dilutions that yield PCR products in no more than 30% of wells should contain one amplifiable cDNA template per positive PCR more than 80% of the time[13]. In our study, only 67% of samples contained a unique genome. This difference could be explained by the nested PCR performed during the SGA step, which could be responsible for artefactual mutations caused by the DNA polymerase, despite our choice of a high fidelity proofreading enzyme. Here, we applied stringent criteria for the selection of clonal samples. The proportion of samples containing one amplifiable cDNA template would have increased from 67 to 74% if we had included the ambiguous samples.

Some positions classified as ambiguous after NGS analysis, showed unexpected double peaks in the Sanger electropherograms profiles (Table 4). Neither of the two techniques was able to place these samples in a defined category. Samples classified as ambiguous should be rejected for functional analysis.

In conclusion, NGS circumvented many difficulties encountered with Sanger sequencing after SGA. NGS does not require the design of specific sequencing primers and spares valuable DNA. In addition, for our purpose, NGS (around 46$ per sample) is more economical than Sanger sequencing (about 77$ per sample).

Finally, this work also demonstrates that NGS of long viral RNA fragments allows more reliable selection of clonal samples obtained by SGA, which is crucial for the study of their functional properties using *in vitro* models.
